# Concordance networks and application to clustering cancer symptomology

**DOI:** 10.1371/journal.pone.0191981

**Published:** 2018-03-14

**Authors:** Teague R. Henry, Sarah A. Marshall, Nancy E. Avis, Beverly J. Levine, Edward H. Ip

**Affiliations:** 1 Department of Psychology and Neuroscience, College of Arts and Sciences, University of North Carolina at Chapel Hill, Chapel Hill, North Carolina, United States of America; 2 Department of Biostatistical Sciences, Wake Forest School of Medicine, Winston-Salem, North Carolina, United States of America; 3 Department of Social Sciences and Health Policy, Wake Forest School of Medicine, Winston-Salem, North Carolina, United States of America; University of South Carolina, UNITED STATES

## Abstract

Symptoms of complex illnesses such as cancer often present with a high degree of heterogeneity between patients. At the same time, there are often core symptoms that act as common drivers for other symptoms, such as fatigue leading to depression and cognitive dysfunction. These symptoms are termed *bridge symptoms* and when combined with heterogeneity in symptom presentation, are difficult to detect using traditional unsupervised clustering techniques. This article develops a method for identifying patient communities based on bridge symptoms termed concordance network clustering. An empirical study of breast cancer symptomatology is presented, and demonstrates the applicability of this method for identifying bridge symptoms.

## Introduction

Cancer survivors may experience a large number of symptoms that persist for years even after active cancer treatment ends [1]. In one study of older breast cancer survivors, the mean number of symptoms reported was 17 [2]. Traditionally, symptoms have been studied and treated in isolation. Recognizing that symptoms may actually be closely intertwined, [3] put forth the idea of the symptom cluster. They defined a symptom cluster as three or more symptoms that tend to occur together and are related in some way.

The nature of the relationship between symptoms may vary across clusters. Symptoms may share a common underlying etiology, perhaps due to the tumor itself (e.g. cytokine induced pain, fatigue, and depression) or an anti-tumor treatment (e.g. chemotherapy induced nausea, vomiting, and altered taste). Alternatively, one symptom in the cluster may actually produce or at least exacerbate the other symptoms (e.g. pain leading to restless sleep, leading to fatigue and concentration difficulties). Even the treatment of a symptom can be linked to other symptoms (e.g. opiates for pain produce fatigue, confusion, and constipation).

Recognition of symptom clusters has the potential to improve symptom management [4, 5]. If one symptom is driving other symptoms within the cluster, targeting that symptom may improve all symptoms. In theory, focusing on the key symptom may be more effective, more feasible, and have fewer side effects than trying to manage all symptoms simultaneously, which would place a heavy burden on the patient. Symptom clusters may also have prognostic value [6]. Specific clusters have been associated with poorer functioning and reduced quality of life; the exact relationship appears to vary according to the symptoms within the cluster [2].

Unfortunately, specific benefits tied to symptom clusters research have been slow to emerge [4]. As of 2016, only 5 studies had tested an intervention designed to manage an entire symptom cluster [7]. Progress has been hampered in part due to a lack of methodological consensus in characterizing clusters. The instruments used to measure symptoms and the statistical methods used to define clusters vary widely, resulting in an extremely heterogeneous list of symptom clusters, even for persons who share the same primary tumor site. A few symptom associations appear to be relatively consistent. In a review of symptom clusters found in advanced cancer patients, [6] noted anxiety-depression, nausea-vomiting, nausea-appetite loss, and fatigue-dyspnea-drowsiness-pain to be most common, whereas [8] noted fatigue/pain, nausea-vomiting, emotional and cognitive clusters were most common.

This conceptualization of a symptom cluster as an intertwined, interacting set of symptoms is closely related to the network psychometrics approach recently pioneered by [9], [10], and [11]. In this perspective, symptoms of psychological disorders are not simply indicators of an underlying disorder, but rather an interacting and possibly self reinforcing manifestation of the disorder that could be driven by one or several common causes. While the underlying causes of a psychological disorder are appreciatively different than the underlying causes of symptoms of cancer, there are several features of the network approach to psychopathology that can be useful in approaching cancer symptomatology.

One core idea to a network approach to symptomatology in general is that of a *bridge symptom*. A bridge symptom, as defined in network psychopathology, is a symptom that is part of multiple disorders [10]. For example, major depression and generalized anxiety disorder have been found to share fatigue, difficulty concentrating and sleep issues, and these are considered to be symptoms that bridge these disorders [10]. These symptoms are thought to be key to understanding the overall structure of individual pathology and comorbidity, as well as early indicators of emerging disorders [12].

While bridge symptoms are used in psychopathology to study commonalities between disorders, their application to cancer symptomatology is less clear. While they could, for example, be used to study commonalities in symptom profiles between cancer types, due to the nature of cancer as a discrete biologically based disease, studying symptom commonalities between cancer types would be less clinically relevant than the equivalent analysis performed on psychological disorders. Instead, this article focuses on bridge symptoms within a cancer type. These bridge symptoms link sets of symptoms within a cancer type together rather than linking symptoms between cancer types.

As an example, consider two symptom profiles that might present in a set of patients with cancer: physical pain and mental issues (difficulty concentrating, mood changes). A patient might present predominantly one or the other set of symptoms, with perhaps a minor amount of overlap. Now consider that the overlap between the symptom sets is consistent, and takes the form of the bridge symptom of fatigue. This symptom links the two symptom clusters of physical pain and mental issues. From a clinical perspective, the identification of fatigue as a bridge symptom is important as bridge symptoms, due to their prevalence in multiple symptom clusters, are more likely to be core features of the underlying disease process. Additionally, analogously to bridge symptoms in psychopathology, bridge symptoms might provide warning to clinicians that a patient is in danger of expressing symptoms from multiple symptom clusters. Consider a patient who has predominantly physical pain symptoms, and who develops fatigue. Due to fatigue’s role in this example as a bridge symptom, the patient is likely more at risk for developing mental symptoms. For these reasons, the identification of bridge symptoms in cancer symptomatology would help shed light on the central effects of the underlying disease process, as well as assist physicians in making targeted treatment programs.

However, there are several issues with studying bridge symptoms in cancer. The first is that unlike in the study of bridge symptoms in psychological disorders, we do not have several disorders that we are trying to find the bridge symptoms for. Instead we have, potentially, symptom clusters that are unobserved and must be inferred. The second issue and one both more salient to the study of bridge symptoms is that there might be heterogeneity of bridge symptoms between clusters. While for one group of patients, the symptom clusters of physical pain and mental issues might be bridged by fatigue, a different group of patients might bridge the *same* symptom clusters with restless sleep. This suggests that there are two conceptual axes along which patients can vary. They can vary in their symptom clusters, and they can vary in what bridge symptoms present.

These dual axes pose more methodological challenges for analyzing bridge symptoms, that of *patient heterogeneity*. One key issue with understanding symptomatology (of any sort, psychological or physical) in patient populations is that symptom expression is often highly heterogeneous for certain illnesses. While it might be the case that every patient with the common cold presents a small set of the same symptoms, patients undergoing cancer treatment or who suffer from a psychological disorder can have differing symptom expression. This notion of patient heterogeneity raises the question of how to group patients so that they share a common symptom network, rather than estimate an averaged symptom network that might comprise a heterogeneous sample, and therefore represent no patient in particular. While commonly used methods for clustering patients based on symptoms, such as hierarchical and other methods of clustering, are used to divide patients into groups based on common symptom sets [13, 14], they do this primarily based on *first-order information*, such as the values of symptom measures and descriptive summaries such as mean and median, and only implicitly include second order information, which refers to the patterns of *co-occurrence of symptoms*. This limits the utility of these methods for forming patient subgroups based on bridge symptoms. What one would obtain from these first order information clustering methods, such as hierarchical clustering or even parametric clustering methods such as latent class analysis [15], are groups of patients that share a similar symptom profile rather than groups of patients who show overlapping symptom clusters linked by common *bridge symptoms*. This is a subtle difference, but one of vital importance. A first order clustering technique would retrieve groups of patients who all experience similar symptoms, dividing patients, for example into our previously described physical pain group and mental symptoms group. However, these clustering techniques would not provide any insight into potential heterogeneity in bridge symptoms. A second order clustering method instead has the potential to retrieve groups of patients that might not share the same overall set of symptoms, but are linked via important bridge symptoms. For example, the first group of patients might contain individuals in the physical pain symptom cluster and individuals in the mental symptoms cluster, with those clusters linked by fatigue, whereas the second group of patients would be characterized by the bridge symptom of restless sleep. Within each group both symptom clusters are expressed, but the differences in bridge symptoms are identified. Finally, identifying bridge symptoms independently of identifying symptom clusters avoids the presupposition that symptom clusters exist at all. This suggests that bridge symptoms can exist where there are no discrete symptom clusters. In that case, the bridge symptoms are not connecting symptom clusters, but rather are representing common, highly salient symptoms in a patient population that is extremely heterogeneous in its symptom expression.

The primary methodological challenge addressed in this article is the development of a method that characterizes groups of patients using not only symptom prevalence, but bridge symptoms as well. To that end, we develop a clustering pipeline termed concordance network clustering that explicitly takes co-occurrence of symptoms, what we have termed second-order information, into account when forming patient communities.

The structure of this article is as follows: First, we provide step by step description of the concordance network clustering procedure, which includes the concordance network construction, between-patient similarity calculations, and random walk community detection. This section uses as its motivating application patient symptomatology, but aims at being a guide to applying concordance network clustering to any appropriate dataset. This section is followed by an application of our methodology to the symptoms of a large sample of breast cancer patients. Clinical implications of the results of the concordance network clustering are discussed and a summary of substantive findings presented. An additional simulation study examining the performance of concordance network clustering can be found in the Supplementary Material (S1 File) for interested readers.

## Concordance network clustering pipeline

The goal of the concordance network clustering pipeline presented here is to group patients into communities that are distinguished by differences in symptom network structure, specifically differences in bridge symptoms. To accomplish this, we implement clustering not on the presence or absence of symptoms, but rather on a representation of the co-occurrence of symptoms within an individual, the concordance network. Once these concordance networks are grouped into patient communities, we use the tools of network psychometrics to describe and interpret the structure of each patient communities’ symptom network. This allows us to identify bridge symptoms, as well as other highly central symptoms that should be taken into account when implementing treatment regimens. Due to the multiple uses of the terms community, cluster and network, we define our terminology here:

- *Concordance network*—A network representation of a *single* patient’s symptoms.
- *Patient community*—a group of patients that share similar bridge symptoms. These groups are the product of concordance network clustering.
- *Patient community concordance network*—a network representation of the symptoms within a patient community.
- *Symptom cluster*—A set of symptoms that co-occur frequently within a specific patient community.

The concordance network clustering pipeline developed here can be divided into five discrete steps:

1. Concordance network construction.
2. Calculating between patient similarity.
3. Clustering patients into communities.
4. Aggregating concordance networks in patient community concordance networks.
5. Computing symptom clusters and describing bridge symptoms.

### Concordance network construction

A *concordance network* is a representation of the co-occurrence of a set of binary variables, in our case symptoms for a single observation of a single subject. It is a matrix with *k* rows and columns, where *k* is the number of symptoms under study. Each entry is 0/1, with 1 representing presence or co-occurrence. The diagonal of the matrix represents the presence or absence of a given symptom, while off-diagonal elements represent the co-occurrence of the symptoms. Note that there is a one-to-one relationship between a concordance network and the original data vector, so no additional information is being added. A concordance network can be easily calculated using the following formula *XX*^*T*^, where X is the original vector of symptoms (0/1), and *T* is the transpose operator. This transformation from vector to matrix serves to emphasize increasing numbers of co-occurring symptoms. If, for example, three symptoms occur in two patients, under a vector expression, the similarity between the two patients is 3, whereas the similarity between the concordance matrices is 6. This similarity scales in an increasing fashion for every additional co-occurring symptom.

**Fig 1:**
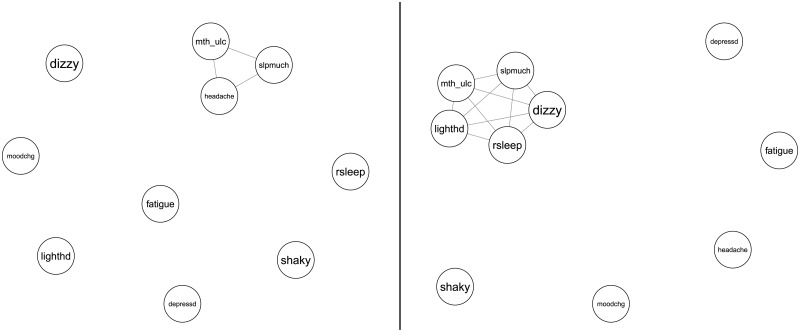
Example concordance networks (Left: Patient A, Right: Patient B). Patient A has co-occurrence of Mouth Ulcers, Headache and too much Sleep, while Patient B has co-occurrence of Mouth Ulcers, Sleeping too much, Light Headedness, Dizziness, and Restless Sleep.

This representation has several advantages over simply having a vector of symptom responses. Concordance networks explicitly show *second order* information, and when used in further analysis more emphasis is placed on the co-occurrence of symptoms than the level of symptoms. This has natural applications to the ideas of symptom clustering. Fig 1 shows two examples of individual concordance networks of simulated patients A and B. We use a subset of 10 of the 39-item symptom list used later in our analysis to illustrate concordance networks. On the subset of symptoms, patient A checked off 3 symptoms—mouth ulcer (mth_ulc), sleeping too much (slpmuch), and headache; patient B checked 5—mth_ulc, slpmuch, dizziness (dizzy), restless sleep (rsleep) and light-headedness (lighthd). Symptoms like shakiness (shaky) is neither checked by patient A nor B and are depicted as “isolates” nodes. The full symptom list and the abbreviations are included in Table 1 in the empirical example.

**Table 1:** Symptom text and abbreviations.

Symptom	Abbreviation
Fatigue or low energy levels	fatigue
Mouth ulcers	mth_ulc
Restless sleep	rsleep
Sleeping too much	slpmuch
Nervousness or shakiness inside	shaky
Mood changes	moodchng
Feeling depressed	depressd
Lightheadedness when standing up	lighthd
Faintness or dizziness at rest	dizzy
Headaches	headache
Swelling of ankles or feet	feetswll
Diarrhea	diarrhea
Nausea	nausea
Constipation	constipa
Abdominal pain/cramps	abdpain
Vaginal dryness	vagdry
Muscle pain/ache/or cramp	muscpain
Weight gain	wgtgain
Weight loss	wgtloss
General aches and pains	genaches
Hot flashes	hotflash
Joint pains	jpains
Night sweats	nsweats
Aches in back of neck and skull	ns_aches
Forgetfullness	forget
Difficulty concentrating	diffconc
Increased appetite	incapp
Short temper	shtempr
Decreased efficiency	decreff
Loss of interest in work/activities	lossint
Lowered work performance	lowperf
Blind spots, fuzzy vision	fuzzyvis
Breast sensitivity/tenderness	brstsens
Avoidance of social affairs	avoid
Cold sweats	csweats
Decreased appetite	decrapp
Feeling of suffocation	suff
Difficulty healing	diffheal
Bloating	bloat

### Calculating between patient similarity

In general, the clustering of any sort of data has three steps. In concordance network clustering the data are the set of concordance networks. The second step is to construct a distance (or similarity) matrix out of the data. The way this distance matrix is constructed determines the nature of the clustering.

The definition of the distance matrix for the clustering of concordance networks is the chief methodological challenge in this article. A concordance network is high dimensional data object with binary (0/1) entries. The challenge with clustering any high dimensional data object is that as the number of dimensions increase, each point becomes more separated, which leads to more uniform distances between points [16]. This renders clustering difficult, as any clustering algorithm relies on non-uniform distances between points.

Our solution to the high-dimensional nature of the concordance networks is to use a similarity measure that adjusts for agreement by chance. Specifically, we use the Adjusted Hubert-Arabie RAND index (ARI; [17]). This measure is used to assess the agreement between two vectors of categorical data, and is commonly used to assess the agreement between clustering algorithms. This ARI adjusts for the agreement by chance, and is calculated based off of the contingency table between two vectors.

The Adjusted RAND index adjusts for the expected value of agreement between two vectors of nominal (such as the labeling of communities, or in our case the entries of the concordance networks) data and can take values typically between 0 and 1, while occasionally it can take negative values. Negative values of the ARI denote that the agreement between two nominal variables is worse then what is expected if the nominal variables were drawn completely at random. This phenomenon is rare, and happens as a consequence of finite sample sizes, as for large samples and asymptotics, the adjustment used by the ARI becomes more and more accurate. The definition of ARI bears resemblance to that for Cohen’s kappa [18], a commonly used metric for agreement between two raters, in which the numerator is the difference between the observed numbers of agreements and the number of agreements expected by chance, whereas the denominator is the total number minus the number of agreements expected by chance. Here, we use the ARI to calculate the agreement between two individuals unique entries on their concordance matrix. By calculating this for every pair of individuals, we create a similarity matrix. After the similarity matrix is constructed, negative entries are set to 0, to reflect that worse agreement than chance can be interpreted as complete lack of agreement. Again, this occurs rarely and as a consequence of small sample sizes.

### Clustering patients using random walk community detection

To determine which concordance networks are similar to one another, we use a community detection approach on the similarity matrix. Community detection approaches [19] belong to a family of algorithms that are used to cluster network data, and recently have been validated on correlation matrices as well as other dense weighted networks (networks with every edge present and real valued) [20]. It needs to be emphasized that the community detection algorithm clusters individual patients’ concordance networks and identifies different subpopulation-level proper networks, which are here called patient communities. We use the random walk community detection method [21]. This community detection method has been shown in simulation studies to perform well on dense weighted undirected networks such as correlation matrices as well as distance or similarity matrices [20]. This algorithm partitions our set of concordance networks into *communities*, within which concordance networks can be thought of as more similar to one another than they would be between communities.

### Aggregation into community concordance networks

After the community detection algorithm is applied, we have community membership information for each concordance network in our sample. The goal then is to *aggregate* concordance networks within a community into a *normalized community concordance matrix* which can then be analyzed using methods from network psychometrics.

**Fig 2:**
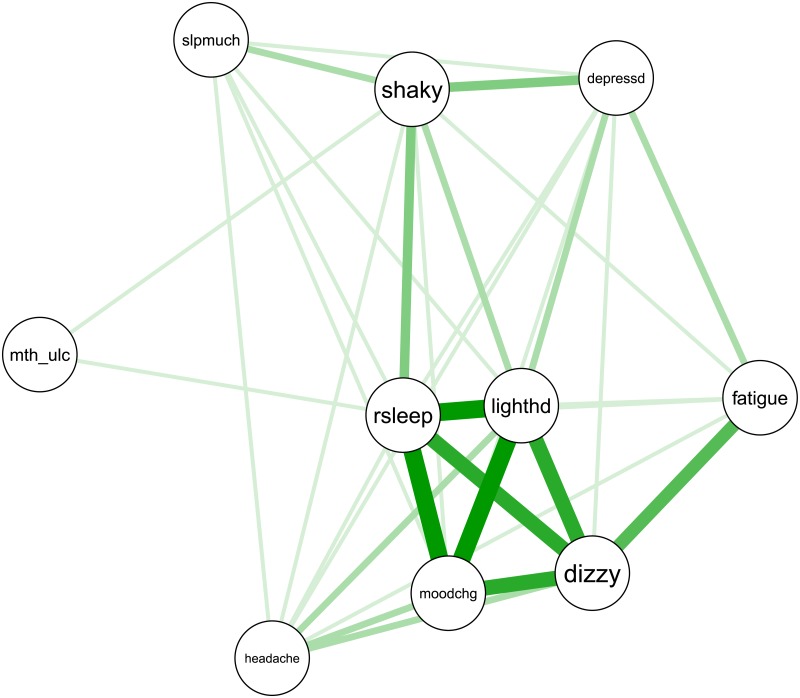
Example community concordance matrix. Thick lines indicate greater co-occurrence of the symptoms in the community. In this case, restless sleep, mood change, dizziness and light headedness have the highest rate of co-occurrence in this simulated community.

In this manuscript, we computed a normalized patient community concordance matrix by summing the concordance matrices within each inferred community, and normalizing each off diagonal element by dividing by the square root of the product of the row and column diagonal element associated with off diagonal element. This creates a metric from 0 to 1, where 1 indicates that, for example, the two symptoms co-occur in every patient in that community. Fig 2 contains an example of a normalized community concordance matrix.

### Characterizing community group concordance networks

After preprocessing and community detection applications, we arrive at a set of community level concordance networks that represent the co-occurrence of some sort of binary set of variables. In this case, as our empirical study analyzing breast cancer symptomatology, these community concordance networks represent symptoms in a set of patients that are similar to each other. To characterize the patient communities, we use the methodology developed by network psychometrics [22] to interpret the structure of each patient communities’ symptom concordance network. In this application, we focus on *bridge symptoms*. Bridge symptoms, as defined in the psychological literature, are symptoms that connect two or more different disorders, such as sleep problems being a part of both major depressive disorder and generalized anxiety disorder [22]. In the present setting of cancer symptoms, bridge symptoms can also be considered symptoms that link two sets of co-occurring symptoms, or if there are no symptom clusters present, linking patient specific symptom profiles.

To determine bridge symptoms, as well as other important symptoms, we calculate several network statistics. We calculate *betweenness*, which is a direct measure of how bridge like a symptom is. In addition to betweenness, we also calculate strength and closeness, two overall measures of how central to the symptom network a particular symptom is. *Strength* is a statistic related to the co-occurrence of a given symptom with other symptoms, while *closeness* is a form of generalized strength, and examines how the symptom is embedded within the whole symptom network. Closeness and strength in weighted dense networks are often very similar in value. We summarized the network statistics below:

- *Betweenness Centrality*—This quantity measures the importance of a symptom as the common factor between multiple other symptoms. It is calculated as the mean of the shortest paths that pass through that symptom. This is our primary metric to decide on bridge symptoms within a cluster [23]. In our analysis, betweenness centrality is standardized (into a z-score) across all compared networks, allowing for comparison.
- *Strength*—This quantity measures how strongly connected a symptom is to all other symptoms. It is calculated as the sum of all paths connected to the symptom [24]. As a measure of centrality, symptoms with higher strength can be thought of as core symptoms to patient’s experience, while lower strength indicates more peripheral symptoms reported by fewer patients. In our analysis strength is z-score standardized.
- *Closeness*—This quantity measures how centrally connected a symptom is to all other symptoms. It is calculated as the inverse of the average shortest path from one symptom to all other symptom [25]. As our concordance networks were fairly dense, closeness has very similar values to strength. Closeness and strength diverge as networks become more sparse. The interpretation of high and low closeness follows the interpretation of strength. In our analysis closeness is z-score standardized.

Once patient communities were estimated, we labeled them based on their bridge symptoms, given these symptoms’ importance in determining intervention strategy. We identified bridge symptoms by examining the relative betweenness of symptoms across the patient communities. If, for example, fatigue, had a far higher betweenness in the first community than in the second, we would label fatigue as a primary bridge symptom in the first community. It is important to note that the labeling of bridge symptoms (or highly central symptoms) is not based on any statistical testing. This is due to two reasons. The first is that the network statistics are descriptive of the derived patient communities, and so provide important information about the structure of those patient communities even in the absence of formal statistics. The second reason is that comparing the values of these network statistics between patient communities is similar to comparing two individuals with regard to height, rather than comparing two groups with regard to their *height distributions*. Whereas if we were comparing height distributions, statistical testing would be warranted, as we are comparing values between “individuals” (i.e. our patient community concordance networks), observing differences in magnitudes is sufficient. For these reasons, our labeling and description of important bridge and central symptoms are based off of these differences in magnitude, and we categorize bridge/highly central symptoms as those with network statistics 2 standard deviations above the mean. These classifications are meant to be descriptive rather than decisive.

Once bridge symptoms are identified using betweenness, we can further interpret how prominent a bridge symptom is by examining relative differences in strength and closeness. If a bridge symptom has high strength and closeness relative to other symptoms, this suggests that the bridge symptom not only links sets of symptoms together, as well as co-occurs quite often with a large number of other symptoms. This sort of highly central bridge symptom is likely a prevalent symptom seen across a variety of patient populations and makes an ideal, albeit obvious target for intervention. Contrast this highly central bridge symptom with a bridge symptom that has average or low strength and closeness. These bridge symptoms link sets of symptoms together, but might not co-occur with a large number of other symptoms. Bridge symptoms with average to low centrality could be viewed as hidden bridge symptoms, as while they likely indicate a underlying disease process due to their nature as bridge symptoms, they are reported less often and co-occur infrequently. In either case, examining bridge symptoms with respect to their relative strength and closeness scores allows researchers to assess the potential impact of a targeted treatment regimen. Centrality plots and calculation of centrality measures were performed with the qgraph package [26] in the R scripting language [27]. Network plots were produced with Gephi [28].

Finally, we compute within patient community symptom clusters using random walk community detection, now applied on the patient community normalized concordance networks. While the discovery and description of symptom clusters is not the focus of this manuscript, it is important to note that concordance network clustering produces patient communities each with potentially unique symptom clusters. These clustering solutions can be evaluated using *modularity*, a quantity that measures how separated nodes in a network are into the specified clusters [29]. Modularity is a relative quantity that scales with the metric of the network, so no absolute thresholds should be used to assess quality of the clustering solution. However, as each patient community concordance network is normalized to be on the same scale, values of modularity can be compared between the patient communities, to obtain a relative idea of the level of separation of symptom clusters.

## Breast cancer symptomatology: An empirical example

### Sample

The data for this application were drawn from an observational study that focused on symptoms and symptom progression among women aged 25 and older who were newly diagnosed with stage I, II or III breast cancer. Data were collected from Memorial Sloan Kettering Cancer Center and the University of Texas-Southwestern Center for Breast Care, with followup questionnaires collected by Wake Forest School of Medicine. All collection sites obtained approval from their Institutional Review Boards. A detailed sample description is available from the original publication [30]. 653 patients were initially recruited, and baseline symptom questionnaires were collected. Followup questionnaires were administered 3, 6, 12, and 18 months after completion of the baseline questionnaire. Of the 653 subjects, 565 completed questionnaires at all time points. However, missing values occurred in their responses. We restricted our analysis to the baseline time point. At baseline, participants were 0–8 months post diagnosis. Of these 653 patients, 531 said that at least one symptom on the symptom questionnaire impacted functioning. Patients who endorsed no symptoms (or no symptoms that impacted functioning) were removed from subsequent analysis, as they form an *a priori* patient community that is not of interest to understanding co-occurrence of symptomatology. Derived patient community concordance networks can be found in S3 File. Researchers interested in working with raw symptom data can contact Wake Forest School of Medicine Institutional Review Board and Dr. Nancy Avis to initiate a data access agreement.

### Symptoms

Study participants completed a 39-item symptom checklist that included symptoms relevant to breast cancer and treatment based on the Women’s Health Initiative study and adapted from the Breast Cancer Prevention Trial Symptom Scale. These symptoms included fatigue, joint pain, constipation, etc. For all symptoms, participants were first asked whether or not the symptom occurred during the past month, and how it impacted regular functioning (no effect, moderate, severe). For this analysis, we dichotomized the symptom severity on the basis of how it impacted functioning. No symptom and no impact on functioning were coded as 0, while moderate and severe impact on functioning was coded as 1. Table 1 contains a list of symptoms and the short identifier used for each symptom in this paper. A copy of the questionnaire used can be found in S2 File.

## Results

We applied concordance network clustering to the previously described set of 531 individuals. We restrict our analysis to just the symptoms at the baseline time point.

**Fig 3:**
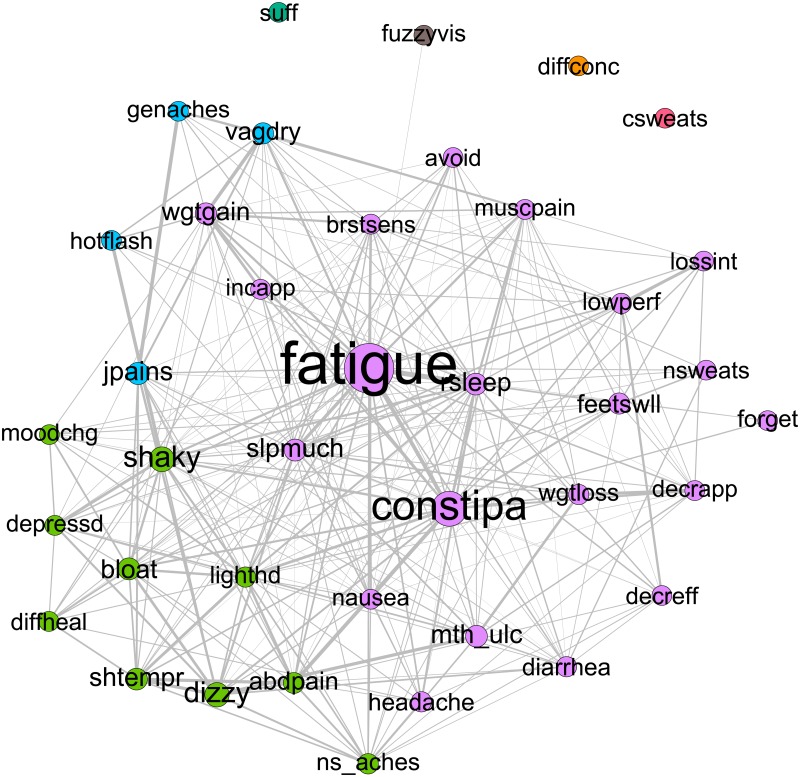
Baseline Fatigue/Constipation community network. Node size is proportional to betweenness. Thicker edges denote higher levels of co-occurrence.

**Fig 4:**
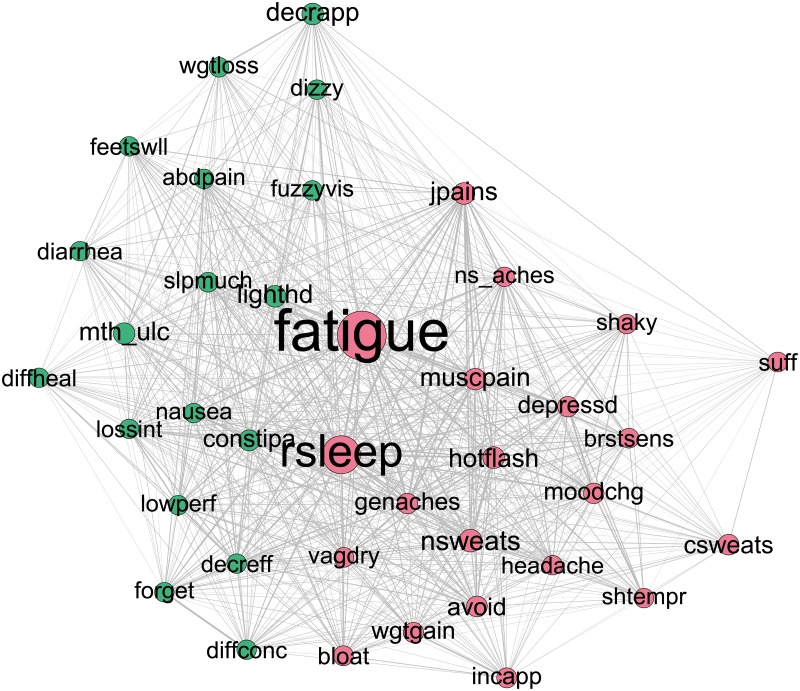
Baseline Fatigue/Sleep community network. Node size is proportional to betweenness. Thicker edges denote higher levels of co-occurrence.

**Fig 5:**
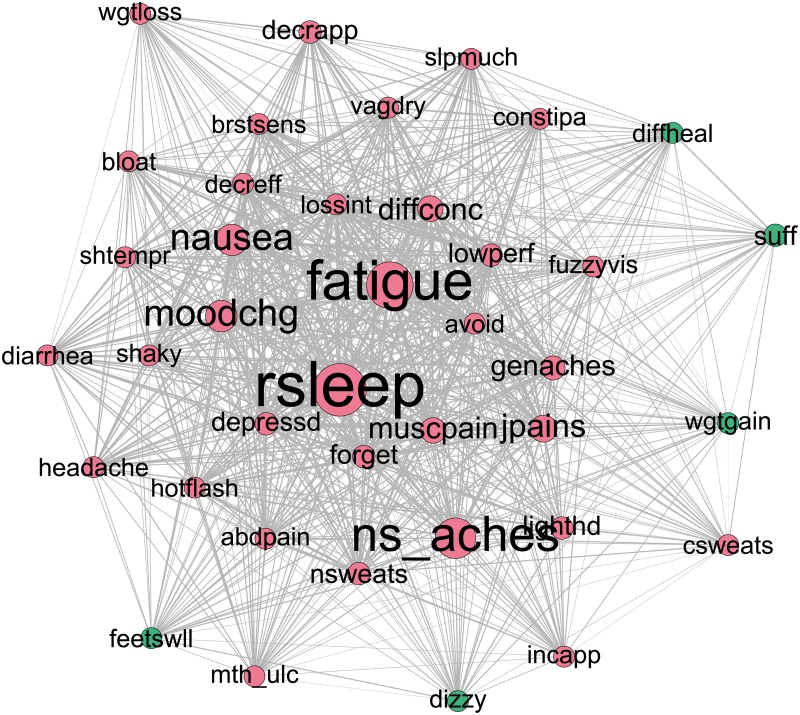
Baseline Fatigue/Sleep/NS Aches community network. Node size is proportional to betweenness. Thicker edges denote higher levels of co-occurrence.

At the baseline time point the concordance network clustering procedure found that the majority of individuals were grouped into one of three large communities, and a small minority of individuals were either grouped into small communities, or were in their own community (an “isolate”). Individuals who were in one of the smaller communities or unique communities endorsed experiencing one or two symptoms in total, and so were highly dissimilar to patients with a large number of symptoms. There were 4 of these smaller communities present, with 20, 7, 5 and 3 patients respectively. Two patients were placed in unique communities. Because of the small sizes of the other communities, we only include the three major communities for presenting the results. Visually, the network for each community can be represented by a graph of nodes of different sizes (representing different levels of an attribute such as betweenness) and edges of different thickness (represent different levels of an attribute such as co-occurrence). Figs 3–5 respectively show the symptom cluster structures for the three identified patient communities at baseline. The size of a node representing a symptom is proportional to its betweenness, and the thickness of an edge indicates the level of co-occurrence between the nodes joined by the specific edge. The first community (Fig 3) is characterized by the bridge symptoms of fatigue and constipation, and is labeled Fatigue/Constipation. The second community (Fig 4) is characterized by fatigue and restless sleep (rsleep) and is labeled Fatigue/Sleep. The third community bears some resemblance to the second community, in that fatigue and restless sleep are prominent bridge symptoms; however neck and spine pain, nausea and mood changes appear to be bridge symptoms as well. As such, this community is labeled Fatigue/Sleep/Neck Spine (NS) Aches. Prevalence and community size for baseline communities, as well as modularity, are presented in Table 2.

**Table 2:** Baseline symptom community size and prevalence.

Community	*n*	%	Modularity
Fatigue/Constipation	141	26.5	.135
Fatigue/Sleep	249	46.8	.049
Fatigue/Sleep/NS Aches	104	19.8	.007

**Fig 6:**
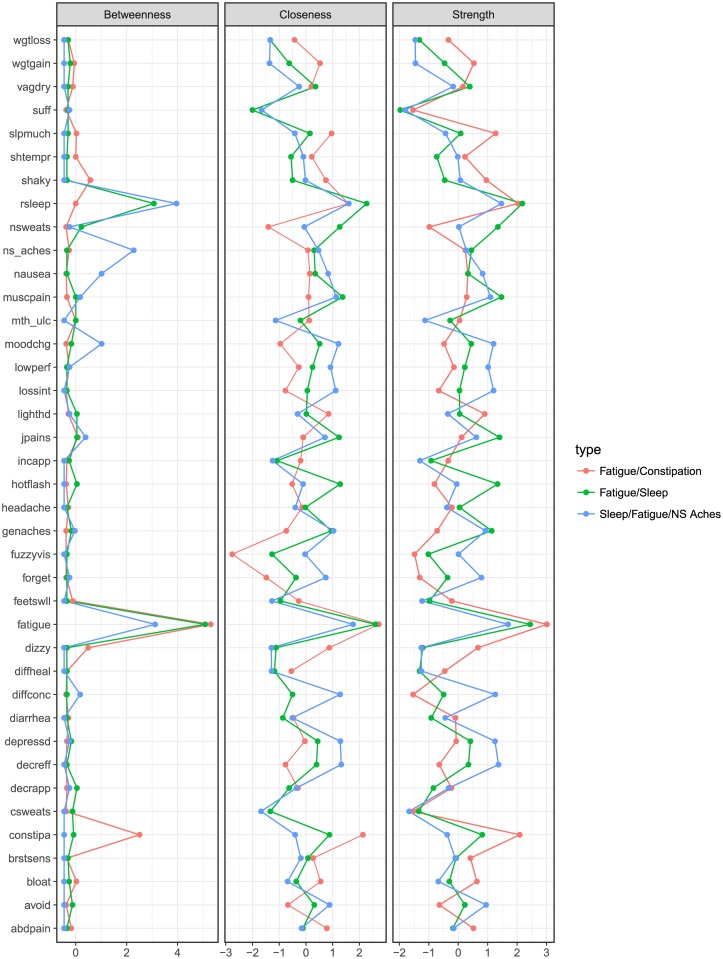
Baseline patient communities centrality values.

To further verify the interpretation of the communities, in Fig 6, we present the plots of the graph theory metrics—betweenness, closeness, and strength—of the symptoms across the communities.

The most prominent, in terms of number of patients, patient community was that of the Fatigue/Sleep community. Here, the dominant and indeed only bridge symptoms present were fatigue and restless sleep, indicated by high values of betweenness relative to other symptoms. These two symptoms had high strength and closeness, each having scores approximately two standard deviations above the mean. This indicates that fatigue and restless sleep are highly central bridge symptoms, with the majority of the patients within this community endorsing them. Additionally, this community had two symptom clusters, with symptoms being approximately evenly divided between the two symptom clusters. The main cluster, to which fatigue and restless sleep were assigned, notably contains a large number of pain related symptoms (muscle pain, neck and spine pain, genital pain) as well as the vasomotor symptoms (night sweats, hot flashes). The second symptom cluster is notable in that it includes all of the gastro-intestinal symptoms (constipation, abdpain, diarrhea, mouth ulcers, nausea). This symptom cluster solution had a modularity value of .049, less than half of the modularity of the Fatigue/Constipation community, suggesting a less separated symptom cluster solution than is found in the Fatigue/Constipation community.

The second most prominent patient community was that of Fatigue/Constipation. This community retained fatigue as its most prominent bridge symptom, with a betweenness score above 4 standard deviations above the mean. Furthermore, the symptom of constipation was the other bridge symptom, with a betweenness score 2 standard deviations above the mean. The symptom clusters showed the greatest amount of separation between the patient communities, with a modularity value of .135. Symptoms were clustered into 3 main groups. Both bridge symptoms were contained in the main symptom cluster, which contains a variety of symptoms with no clear theme. The next largest symptom cluster (green in Fig 3) contained mood related symptoms such as depression, mood change and short temper, among other non-mood related symptoms. Finally, the third main symptom cluster (blue in Fig 3) contains the related symptoms of hot flashes, vaginal dryness and genital pain, along with joint pain. Finally, the strength and closeness profiles show that fatigue and constipation are also highly central symptoms (strength and closeness over 2 SD away from the mean) that co-occur commonly with a variety of other symptoms. An additional highly central symptom, yet one that is not classified as a bridge symptom is that of restless sleep. This suggests that while restless sleep co-occurs on average with the same number of other symptoms as constipation, constipation co-occurs with symptoms from different symptom clusters more often than restless sleep.

The final community, and the least prominent, is that of the Sleep/Fatigue/NS Aches community. This community is distinguished from the Fatigue/Sleep community by the bridge symptom profile. Restless sleep had a higher value for betweenness than fatigue, and neck/spine aches also achieved the threshold for classification as a bridge symptom. Two symptom clusters were estimated, with a modularity value of .007. This modularity value was seven times lower than the next highest modularity, suggesting that the symptom clusters were not well separated by comparison. This is borne out by inspection of the symptoms in each cluster, with the vast majority of symptoms being in the first cluster, and four symptoms being included in the second cluster. This suggests that the symptom clusters found here are not necessarily relevant, and that we can effectively treat this community as a single symptom cluster. This changes our interpretation of the bridge symptoms. Instead of potentially linking disparate symptom clusters, these bridge symptoms can be interpreted as linking patient specific symptom profiles. Patients in this community might not show a great deal of homogeneity in their symptom presentation, but they are more likely to express the symptoms of fatigue, restless sleep and neck/spine aches. Additionally, no symptom exceeds our cut-off for classification as highly central, suggesting a substantial amount of heterogeneity in symptom presentation within this community.

## Discussion

The concordance network clustering approach groups patients into communities based on both the bridge symptoms that characterize that community of patients as well as the closeness and strength as secondary indicators of community. As was mentioned above, these bridge symptoms might be the cause of other exhibited symptoms, and prime targets for treatment. The defining of the patient communities primarily by bridge symptoms (as opposed to closeness or strength) is an advantage to concordance network clustering.

Given that all three baseline communities revealed fatigue to have high betweenness-centrality, fatigue might be an ideal early target for symptom management. Furthermore, the differences between the three patient communities in terms of both bridge symptoms, but also symptom cluster structure suggest several specific avenues of treatment. For example, the higher modularity solution of the Fatigue/Constipation patient community, combined with the clear set of bridge symptoms, suggests that both a general treatment of bridge symptoms and individualized treatment for the specific symptom cluster could be effective. This approach would be less effective in the Sleep/Fatigue/NS Aches patient community, where the lack of a clear symptom cluster structure combined with lower variability in centrality measures suggest that patients in this community are fairly heterogeneous in their symptom expression. Here, a treatment that just targets the bridge symptoms, restless sleep, fatigue and neck/spine aches, might be more effective than designing treatment around symptom clusters. The identification of unique bridge symptoms in different communities implies that different management strategies may be needed depending on the subgroup of patients. Therefore, any symptom management plan is likely to require tailoring to the individual patient. An important unresolved issue is deciding which community most accurately represents any given patient presenting for care.

As illustrated by the application to the symptom data, the methodology of clustering concordance network has several advantages. First, it uses second-order information for clustering individual networks and form proper networks that can be interpreted. Second, the method is discriminating and is able to lead to distinct communities that exhibit rather different network structures in symptomatology. These patient communities can then be examined for symptom clusters as well as identifying relevant bridge symptoms. Finally, within each community, our pipeline extracts network metric of specific symptoms such as betweenness—this can increase our understanding of the role of specific symptoms within a network.

Future studies could better characterize demographic and treatment-related characteristics of the members of different communities. For example, are members of the community with predominantly cognitive and psychological symptoms mostly older or younger women, or are they heterogeneous in terms of age? The presence and positioning of certain symptoms within the network may also have some prognostic value—for example, fatigue versus pain as the main bridge symptom may portend a better or worse prognosis, although this has yet to be ascertained. Future research could also track the evolution of symptom networks over time in greater detail, i.e. determine patients’ transition rates in an out of networks found at different time points.

The methodology as presented in the article also has some limitations. First, symptom networks tend to evolve over time, and the method does not track individual patients longitudinally. While the study of different communities over time is useful, novel methods will be required to truly delineate symptom evolution through the analysis of individual trajectories. Currently work is in progress to design models for analyzing longitudinal network data along the lines of hidden Markov models [31]. Second, the clustering algorithm can be sensitive to the choice of parameters and metrics. Different similarity measures may lead to different solutions. Thus the robustness of the similarity measures needs to be further investigated. Currently the method applies to dichotomous network variables (presence or absence of function impacting symptoms). Including other types of variables including ordinal and continuous will be a topic for further research. Furthermore, though the simulation study presented in S1 File indicates that concordance network clustering can retrieve patient communities where more traditional methods of clustering cannot, further work needs to be done to validate concordance network clustering as a tool for identifying patient communities of interest. Specifically, evaluating the performance of this method in the *absence* of strong bridge symptoms would be an important next step to determining the scope of applicability.

In summary, as illustrated by the symptom networks derived from a sample of breast cancer survivors, the proposed new method is able to delineate heterogeneity in network structures and identify bridge symptoms for each subgroup of survivors. The method can be readily applied to other individual-level symptom concordance networks across a variety of health domains.

## Supporting information

S1 File Simulation study. A simulation study examining performance of concordance network clustering.
(PDF)

S2 File Symptom questionnaire. The original symptom questionnaire used in this study.
(DOC)

S3 File Group concordance networks. The derived group concordance networks used in this study.
(ZIP)
