# Supplementary material for: Concordance networks and application to clustering cancer symptomology
Source: PLoS One. 2018 Mar 14;13(3):e0191981. doi: 10.1371/journal.pone.0191981 (PMC5851541; doi:10.1371/journal.pone.0191981)
Supplement: S1 File — A simulation study examining performance of concordance network clustering. (PDF) [file pone.0191981.s001.pdf]

S1: Simulation Study

December 14, 2017

Simulations

In this appendix we present a small simulation study examining the performance of concordance network clustering compared to hierarchical clustering. To do this we have generated artificial data that has a dependency structure similar to a concordance structure. In this study, we generate two groups of artificial subjects, each with a set number of symptom items. The first group exhibits dependency in the first half of the symptoms, while the second group exhibits dependency in the second half of the symptoms. Importantly, the marginal probability of any symptom occurring is the same across both groups. A visual depiction of the artificial data is shown in Figure 1 below.

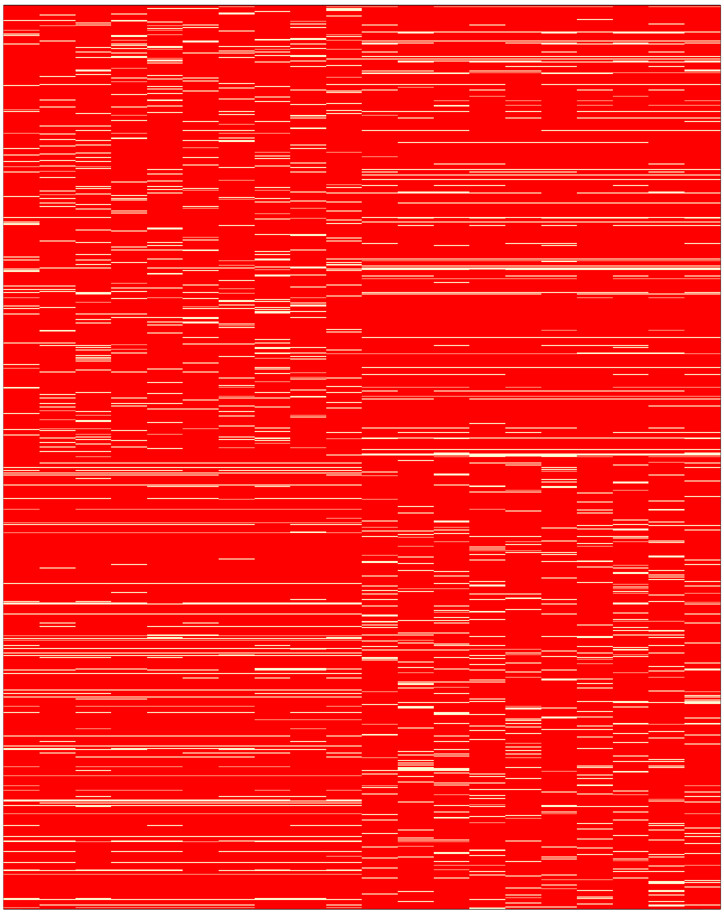

Figure 1: Heatmap of simulated data set. Rows are subjects, columns are "symptom" items. White represents endorsement of symptom. In this simulated dataset, the first half of the subjects exhibit high concordance in the second half of the symptom set, and vice versa for the second half of the subjects. Importantly, marginal rate of symptom endorsement are equal between subject halves.

In this simulation, we manipulate four quantities: total number of subjects n, total number of items n_i, level of dependency between items within a group (quantified as a correlation, ρ), and marginal rate of symptom occurrence p. The specific mechanics of the simulation are as follows:

- Generate $n/2$ samples from a multivariate normal of dimension n_i, mean vector 0, and a covariance matrix with variance of 1 for each dimension, and the first $n_i/2$ items correlated at ρ while all other dimensions are uncorrelated.
- Generate the second group of $n/2$ samples in a similar fashion, except with the last $n_i/2$ items correlated at ρ, with all other dimensions being uncorrelated.
- Threshold the normal covariates at the quantile value that corresponds to the marginal rate of symptom occurrence p. Set items with values above this threshold to 1, and all others to 0.

We had a total of 11 condition sets, summarized in Table 1. In each condition set, 100 replications were generated for assessing performance.

Table 1: Simulation Conditions

#	n	ρ	n_j	p
1	500	.5	10	.1
2	1000	.5	10	.1
3	500	.9	10	.1
4	1000	.9	10	.1
5	500	.5	20	.1
6	1000	.5	20	.1
7	500	.9	20	.1
8	1000	.9	20	.1
9	500	.5	10	.3
10	1000	.5	10	.3
11	500	.9	10	.3
12	500	.5	20	.3
13	1000	.5	20	.3
14	500	.9	20	.3
15	1000	.9	20	.3

Analysis of Simulated Results

For each generated dataset, concordance matrix clustering was applied as described previously. In addition to this, hierarchical clustering was performed using a binary distance metric and a Ward's agglomerative algorithm. Two clusters were extracted from the hierarchical clustering method using the cut-tree approach.

For the concordance network approach, a highly relevant phenomenon was noticed. In every condition, more than two communities were extracted by the concordance network approach. Typically three communities were found. In each case, two of the communities had subjects with a moderate to high number of symptoms, while the rest of the communities consisted of subjects with no symptoms or 1 symptoms. The two informative communities corresponded more tightly to the *a priori* generative groups, while the uninformative communities were distributed throughout the data. This had the effect of lowering classification accuracy when one included the uninformative communities in the comparison to the generative groups. As the uninformative communities can be easily detected in an empirical data setting, we removed the uninformative communities from the assessment of classification accuracy. This phenomena of uninformative communities/subjects occurs whenever the unobserved groupings are based on differences second order information rather than first order information. It corresponds to the example of two observations generated from a two bivariate normal distributions with opposite correlations, and each observation being close to the mean value of $(0, 0)$. In this case, there is no information available to accurately classify those observations.

In the case of the hierarchical clustering, we assessed the clustering pattern when we increased the number of extracted communities. The same phenomenon was not observed at any number

of extracted communities, and increasing the number of extracted communities did not improve classification accuracy. Fig-2 below shows jitter plots of the ARI for the agreement between the true generative groups and the extracted communities from the two assessed methods, concordance matrix clustering with removal of uninformative groups, and the two cluster hierarchical solution.

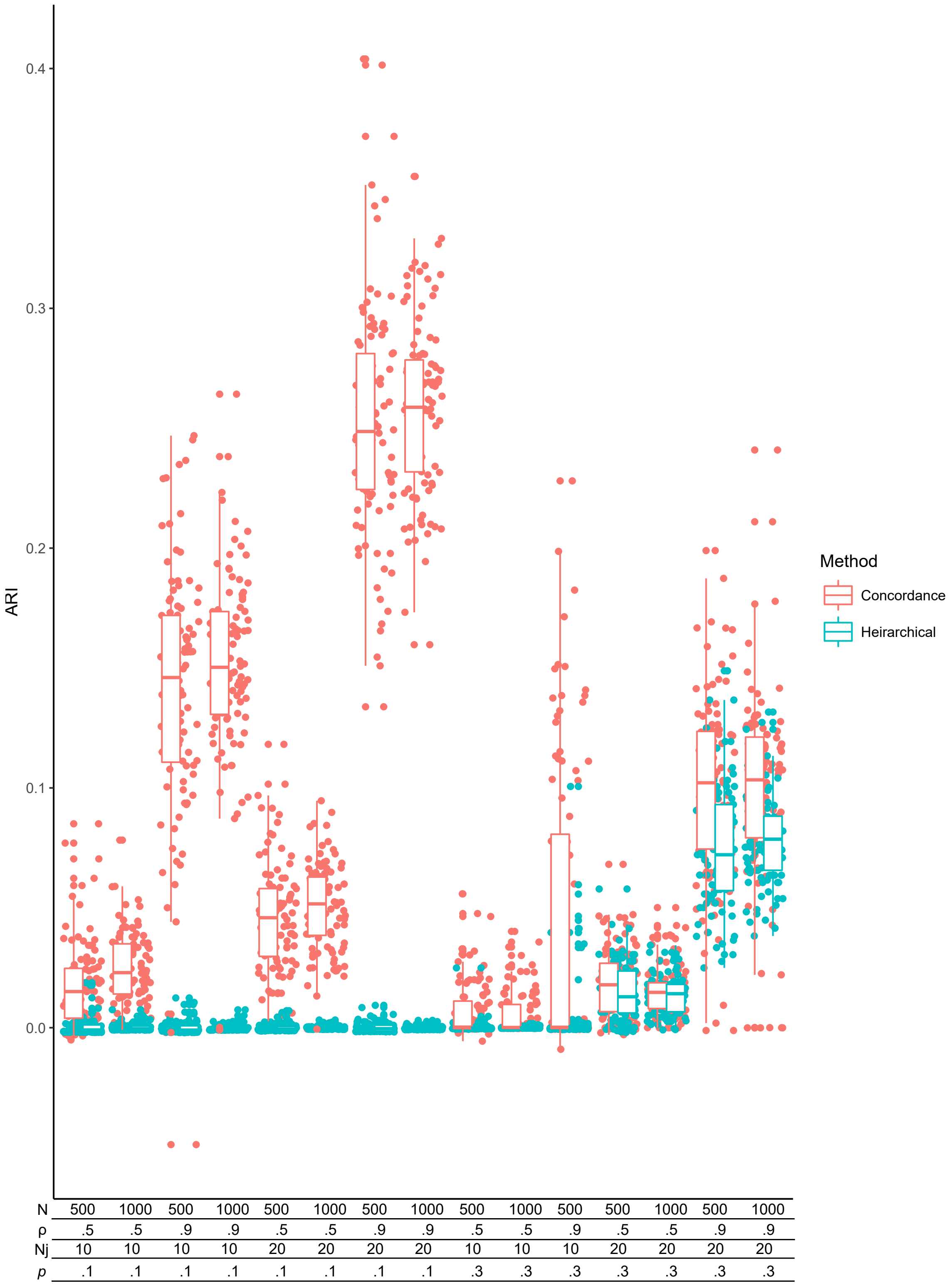

Figure 2: Box and jitter plots of ARI for both the concordance network clustering (with removal of uninformative communities) and the hierarchical clustering. Note that in all cases, concordance network clustering outperforms hierarchical clustering. Note too that the conditions for which hierarchical clustering achieves ARI above 0 are the conditions with the highest marginal probability, as well as the largest number of items.

There are several salient points presented by the results above. The first is that neither method ever achieves a high ARI, with the maximum ARI achieved being around .4. This corresponds to an unadjusted RAND of approximately .70, which suggests an approximate 70% classification accuracy. This finding suggests that the classification of binary data based on second order information differences is a difficult problem, regardless of classification algorithm. The largest simulation factor that effects the ARI results for the concordance network clustering is the level of dependency within groups ρ, with increase in ρ being associated with relatively large increases in ARI for only the concordance network clustering method. Conversely, it appears that a higher marginal base rate decreases the classification accuracy for concordance networks, and increases the classification accuracy for hierarchical clustering, though concordance network clustering still outperforms the hierarchical clustering. As a final point, it should be noted that hierarchical clustering has no classification accuracy in many of the conditions (ARI is approximately 0). This further suggests hierarchical clustering's inability to cluster on second order information.
