# Supplementary material for: Concordance networks and application to clustering cancer symptomology
Source: PLoS One. 2018 Mar 14;13(3):e0191981. doi: 10.1371/journal.pone.0191981 (PMC5851541; doi:10.1371/journal.pone.0191981)
Supplement: S2 File — The original symptom questionnaire used in this study. (DOC) [file pone.0191981.s002.doc]

**Patient I.D.** ___ ___ - ___ ___ ___ ___

**Patient Acrostic** ___ ___ ___ ___ ___ ___

**SYMPTOMS QUESTIONNAIRE**

Below are statements about symptoms some people may experience. For each statement, check the appropriate box for the response that best describes how bothersome the symptom was for you **during the past month**. If you did not have the problem, check the box under the column titled “symptom did not occur.” Please do not skip any questions. **Mark only one box on each line.**

If you experienced the symptom, use the following key to indicate how bothersome it was:

**Mild = symptom did not interfere with usual activities.**

**Moderate = symptom interfered somewhat with usual activities.**

**Severe = symptom was so bothersome that usual activities could not be performed.**

| **Symptom** | **Symptom did not occur** | **Symptom Occurred and Was:** | | |
| --- | --- | --- | --- | --- |
|  |  | **Mild** | **Moderate** | **Severe** |
| 1. Fatigue or low energy level |  |  |  |  |
| 2. Mouth ulcers |  |  |  |  |
| 3. Restless sleep |  |  |  |  |
| 4. Sleeping too much |  |  |  |  |
| 5. Nervousness or shakiness  inside |  |  |  |  |
| 6. Mood changes |  |  |  |  |
| 7. Feeling depressed |  |  |  |  |
| 8. Lightheadedness when  standing up |  |  |  |  |
| 9. Faintness or dizziness at rest |  |  |  |  |
| 10. Headaches |  |  |  |  |
| 11. Swelling of ankles or feet |  |  |  |  |
| 12. Diarrhea |  |  |  |  |
| 13. Nausea |  |  |  |  |

| **Symptom** | **Symptom did not occur** | **Symptom Occurred and Was:** | | |
| --- | --- | --- | --- | --- |
|  |  | **Mild** | **Moderate** | **Severe** |
| 14. Constipation |  |  |  |  |
| 15. Abdominal pain/cramps |  |  |  |  |
| 16. Vaginal dryness |  |  |  |  |
| 17. Muscle pain/ache/or cramp |  |  |  |  |
| 18. Weight gain |  |  |  |  |
| 19. Weight loss |  |  |  |  |
| 20. General aches and pains |  |  |  |  |
| 21. Hot flashes |  |  |  |  |
| 22. Joint pains |  |  |  |  |
| 23. Night sweats |  |  |  |  |
| 24. Aches in back of neck and skull |  |  |  |  |
| 25. Forgetfulness |  |  |  |  |
| 26. Difficulty concentrating |  |  |  |  |
| 27. Increased appetite |  |  |  |  |
| 28. Short temper |  |  |  |  |
| 29. Decreased efficiency |  |  |  |  |
| 30. Loss of interest in work/activities |  |  |  |  |
| 31. Lowered work performance |  |  |  |  |
| 32. Blind spots, fuzzy vision |  |  |  |  |
| 33. Breast sensitivity/tenderness |  |  |  |  |
| 34. Avoidance of social affairs |  |  |  |  |
| 35. Cold sweats |  |  |  |  |
| 36. Decreased appetite |  |  |  |  |
| 37. Feelings of suffocation |  |  |  |  |
| 38. Difficulty healing |  |  |  |  |
| 39. Bloating |  |  |  |  |
